# Robot-Assisted Radical Prostatectomy in Renal Transplant Recipients: A Systematic Review

**DOI:** 10.3390/jcm12216754

**Published:** 2023-10-25

**Authors:** Alberto Piana, Alessio Pecoraro, Flavio Sidoti, Enrico Checcucci, Muhammet İrfan Dönmez, Thomas Prudhomme, Beatriz Bañuelos Marco, Alicia López Abad, Riccardo Campi, Romain Boissier, Michele Di Dio, Francesco Porpiglia, Alberto Breda, Angelo Territo

**Affiliations:** 1 Department of Urology, University of Turin, 10043 Turin, Italy; 2 Department of Urology, Romolo Hospital, 88821 Rocca di Neto, Italy; 3 Department of Experimental and Clinical Medicine, University of Florence, 50134 Florence, Italy; alessio.pecoraro10@gmail.com (A.P.); riccardo.campi@unifi.it (R.C.); 4 Department of Surgery, Candiolo Cancer Institute FPO-IRCCS, Candiolo, 10060 Turin, Italy; 5 Department of Urology, İstanbul Faculty of Medicine, İstanbul University, 34093 İstanbul, Turkey; 6 Department of Urology, Kidney Transplantation and Andrology, Toulouse Rangueil University Hospital, 31400 Toulouse, France; prudhomme.t@chu-toulouse.fr; 7 Division Renal Transplantation and Reconstructive Urology, Hospital Universitario El Clínico San Carlos, 28040 Madrid, Spain; 8 Department of Urology, Virgen de la Arrixaca University Hospital, 30120 Murcia, Spain; 9 Department of Urology and Renal Transplantation, La Conception University Hospital, 13005 Marseille, France; romainboissier@hotmail.com; 10 Division of Urology, Department of Surgery, Annunziata Hospital, 87100 Cosenza, Italy; 11 Unit of Uro-oncology and Kidney Transplant, Department of Urology, Puigvert Foundation, Universitat Autònoma de Barcelona (UAB), 08025 Barcelona, Spain

**Keywords:** robot-assisted radical prostatectomy, renal transplant, recipients, kidney transplantation

## Abstract

Robot-assisted radical prostatectomy (RARP) has been shown to achieve excellent oncological outcomes with a low rate of complications in patients with prostate cancer. However, data on RARP in renal transplant recipients (RT) are dispersed. A literature search was conducted through April 2023 using PubMed/Medline, Embase and Web of Science databases. The primary aim was to evaluate the safety, oncologic and clinical outcomes of RARP in RT recipients. The secondary aim was to identify surgical technique modifications required to avoid iatrogenic damage to the transplanted kidney. A total of 18 studies comprising 186 patients met the inclusion criteria. Age at the time of treatment ranged 43–79 years. Biopsy results showed a high prevalence of low- and intermediate-risk disease. Operative time ranged between 108.3 and 400 mins, while estimated blood loss ranged from 30 to 630 mL. Length of hospital stay ranged from 3 to 6 days whereas duration of catheterization was between 5 and 18 days. Perioperative complication rate was 17.1%. Overall positive surgical margin rate was 24.19%, while biochemical recurrence was observed in 10.21% (19/186 patients). Modifications to the standard surgical technique were described in 13/18 studies. Modifications in port placement were described in 7/13 studies and performed in 19/88 (21.6%) patients. Surgical technique for the development of the Retzius space was reported in 13/18 studies. Data on lymphadenectomy were reported in 15/18 studies. Bilateral lymphadenectomy was described in 3/18 studies and performed in 4/89 (4.5%) patients; contralateral lymphadenectomy was reported in 7/18 studies and performed in 41/125 (32.8%) patients. RARP in RTRs can be considered relatively safe and feasible. Oncological results yielded significantly worse outcomes in terms of PSM and BCR rate compared to the data available in the published studies, with an overall complication rate highly variable among the studies included. On the other hand, low graft damage during the procedure was observed. Main criticisms came from different tumor screening protocols and scarce information about lymphadenectomy techniques and outcomes among the included studies.

## 1. Introduction

Genitourinary tumors have been reported to be the second most common malignancy in the renal transplant recipient (RTR) population in the United States [1]. Among these, prostate cancer (PCa) is the most prevalent [1]. The incidence of PCa in RTRs has not been extensively studied, but the available data indicate an incidence rate ranging from 0.72% to 3.1% [2,3]. Of note, more than half of RTRs are >50 years old and usually enrolled in specific surveillance protocols where they are closely followed up and screened for cancer. Subsequently, a higher incidence of PCa has been reported in RTSs when compared to the general population [4,5]. Moreover, there is still a lack of evidence regarding the potential higher risk of worse oncological and functional outcomes among RTRs. As a result, the best treatment strategy for PCa in RTRs has not been defined yet [6].

Robot-assisted radical prostatectomy (RARP) is the most commonly used approach for the treatment of organ-confined prostate cancer in the general population [7]; however, there is a lack of high-quality evidence on the outcomes of RARP in RTRs. The surgical complexity of the procedure may be increased not only by the presence of the graft in the iliac fossa but also by the development of adhesions at the level of the external iliac vessels as well as the possible unusual course of the ureter. Even though a systematic review to uncover cumulative data on this subject was published in 2018, it only covered articles till 2018 [8], studies with higher patient numbers [9] or multicentric series [10] that had been published in the recent years.

In order to bring out the current data on RARP in RTRs, we systematically reviewed the available evidence on the use of the robotic approach for radical prostatectomy in kidney transplant recipients.

## 2. Materials and Methods

### 2.1. Search Strategy

We conducted a systematic review in line with the Preferred Reporting Items for Systematic Reviews and Meta-analyses (PRISMA) guidelines [11]. This protocol was registered in the International Prospective Register of Systematic Reviews (PROSPERO) database (Registration Number: CRD42023450000). For this study, PubMed, Embase and Scopus databases were searched throughout April 2023 for RARP in RTRs. We used the following search strategy: (“Prostatic Neoplasms/surgery”[Mesh] OR “Prostatectomy”[Mesh] OR prostatect*[tiab] OR (resection[tiab] OR removal[tiab] AND prostat*[tiab])) AND (“Robotics”[Mesh] OR “Robotic Surgical Procedures”[Mesh] OR robot*[tiab] OR Davinci*[tiab] OR “Da-Vinci*”[tiab] OR Senhance*[tiab] OR “Revo-I*”[tiab] OR Versius*[tiab] OR Avatera*[tiab] OR Hinotori*[tiab]) AND (“Kidney Transplantation”[Mesh] OR ((“Kidney”[Mesh] OR kidney*[tiab] OR renal[tiab] OR ephron*[tiab]) AND (allorecipient*[tiab] OR autorecipient*[tiab] OR “transplantation”[Subheading] OR “Transplantation”[Mesh] OR “Transplants”[Mesh] OR transplant*[tiab] OR allotransplant*[tiab] OR autotransplant*[tiab] OR allograft*[tiab] OR autograft*[tiab] OR graft*[tiab] OR recipient*[tiab]))).

Article screening was performed by two authors (A.P. and E.C.) and eventual discrepancies were solved by a third author (A.T.). The primary aims of this systematic review were to evaluate the safety, oncologic and clinical outcomes of RARP. The secondary aim was to identify surgical technique modifications required to avoid iatrogenic damage to the transplanted kidney.

### 2.2. Study Selection

A specific population (P), intervention (I), comparator (C), outcome (O), and study design (S) (PICOS) framework was assessed to define the study eligibility [10]. The PICOS framework for this review was as follows:

- (P): adults (age > 18 yrs) who underwent RARP or modified RARP for prostate cancer after kidney transplantation;
- (I): RARP or modified RARP;
- (C): either comparative or noncomparative studies;
- (O): incidence of positive surgical margins, intra- and postoperative complications and functional outcomes;
- (S): prospective or retrospective studies.

All articles with data of interest were selected; only articles that have a full English text and are pertinent to PICO strategy were included. Abstracts, editorials, commentaries, reviews, book chapters, non-English publications and articles reporting experimental studies on animals or cadavers were excluded.

### 2.3. Data Extraction

**Figure 1:**
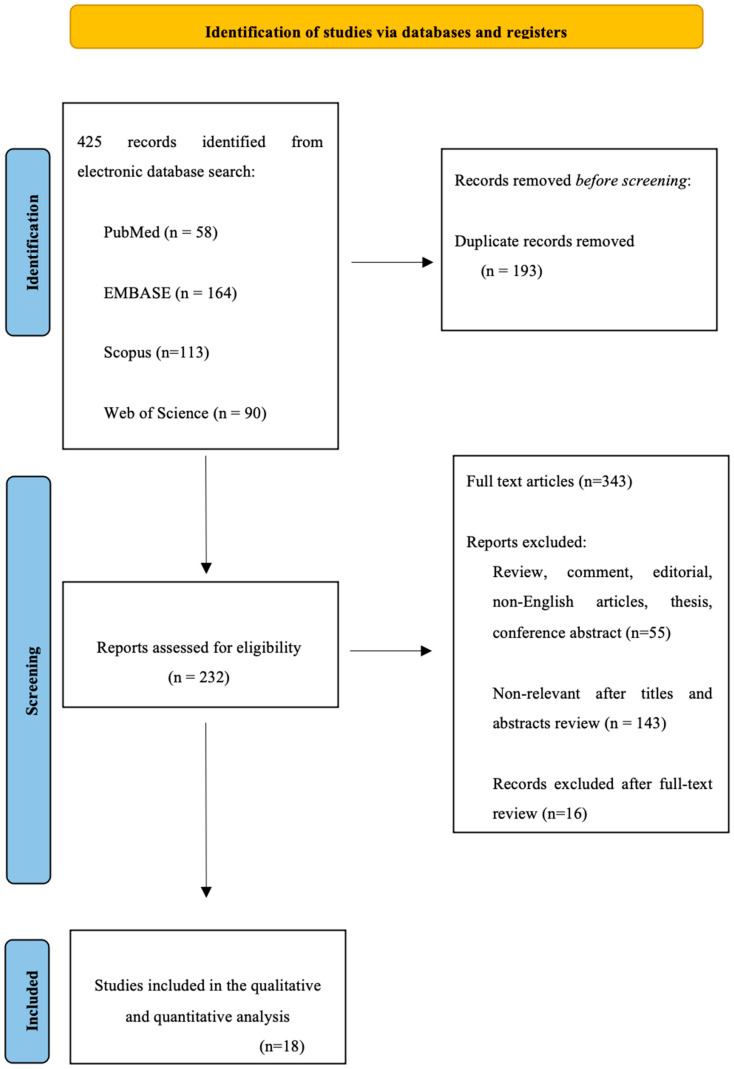
Prisma flow chart—study selection with inclusion and exclusion criteria of the reviewed studies.

The articles were independently reviewed by two of the authors (A.P. and F.S.) on the basis of the inclusion and exclusion criteria. Titles and abstracts were analyzed. After the initial screening, a full-text review was conducted to confirm the eligibility for inclusion. Finally, references from the selected articles were reviewed to identify other possible sources of data. Disagreements regarding study selection were solved by a third reviewer (A.T.) (Figure 1).

### 2.4. Risk-of-Bias Assessment

**Figure 2:**
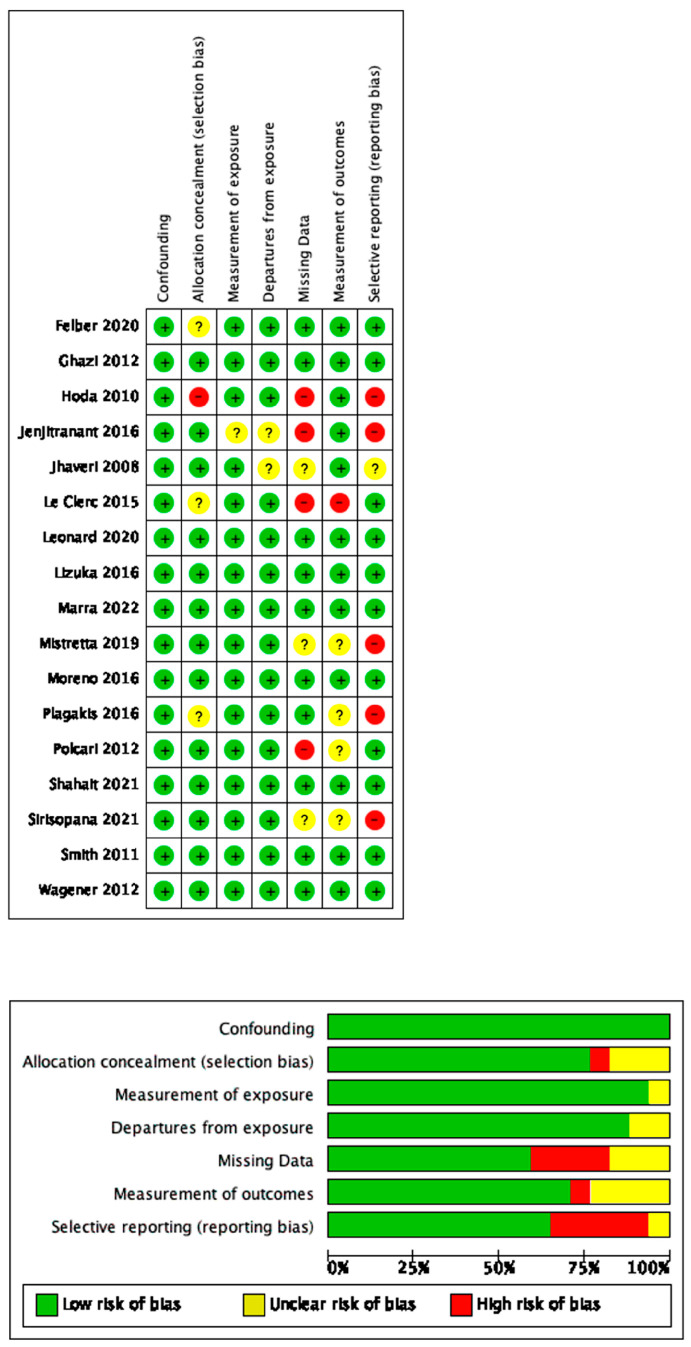
Evaluation of the risk of bias and confounders using ROBINS-I tool for nonrandomized studies.

The risk-of-bias assessment was performed independently by two authors (A.P. and F.S.) using the ROBINS-I tool for nonrandomized studies. Disagreement was solved by a third author (A.T.). The risk of bias was measured over four domains of interest (participants, predictors, outcome and analysis) (Figure 2).

### 2.5. Data Extraction and Analysis

Baseline demographics (age, body mass index, preoperative PSA, etc.), surgical technique details (port placement, development of the space of Retzius, lymphadenectomy), perioperative variables (operating time, estimated blood loss [EBL], complication, length of stay, etc.), oncological outcomes (positive surgical margins [PSM] and biochemical recurrence [BCR]) were extracted from the included studies.

## 3. Evidence Synthesis

### 3.1. Study Characteristics

The literature search (Supplementary Materials) included 425 records. After screening and eligibility assessment, 18 retrospective studies met the inclusion criteria (Figure 1).

Overall, 186 patients were included. Table 1 summarizes the main characteristics of the studies included. Table 2 summarizes the modifications to the standard surgical technique.

### 3.2. Baseline Characteristics

Age at the time of treatment was between 43 and 79 years, while preoperative PSA levels ranged from 6.17 to 130 ng/mL. Prostate biopsy showed a high prevalence of low- and intermediate-risk disease {Gleason 3 + 3 [75/186 patients (40.32 %)], Gleason 3 + 4 [72/186 patients (38.77 %)], Gleason 4 + 3 [31/186 patients (16.66 %)], Gleason 4 + 4 [6/186 (3.22%)] and Gleason 4 + 5 [10/186 patients (5.37 %)]}.

### 3.3. Surgical Technique

Modifications to the standard technique [27] were described in 15/18 studies [8,9,10,12,14,15,16,17,18,19,20,22,23,24,25] We identified three primary steps of the procedure in which technical modifications were made: port placement, development of the space of Retzius and lymphadenectomy.

Port placement template was reported in 16 studies [8,9,10,12,14,15,16,17,18,19,20,21,22,23,25,26]. Modifications in port placement were described in 7/13 studies and performed in 19/88 (21.6%) patients. The surgical technique for the development of the space of Retzius was reported in 13/18 studies [8,12,14,15,16,17,19,20,21,22,23,25,26]. Modifications were described in 9/10 studies and performed in 19/22 (86.3%) patients, including a complete Retzius-sparing approach in 1/10 studies, 4/22 (18%) development from the contralateral side (7/10 studies, 17/22 patients (77%) and an extraperitoneal approach (1/10 studies, 1/22 patients, 4.5%).

Data on lymphadenectomy were given in 8/18 studies including 137 patients [8,9,10,16,17,18,23,24]. Bilateral lymphadenectomy was carried out in 3/8 studies [4/137 (3%) patients] [9,10,23]; lymph node harvesting after bilateral lymphadenectomy was reported in 1/8 studies [23]. On the other hand, 3/19 harvested lymph nodes were found positive at the final pathological analysis in 1/9 patients. Contralateral lymphadenectomy was performed in 5/8 studies [55/137 (40%) patients] [8,9,10,16,17,18,23,24]. Subsequently, lymph node harvesting after contralateral lymphadenectomy was reported in 1/8 studies [23]. Of these, 1/3 harvested lymph nodes were found positive at the final pathological analysis in 1/9 patients. One study reported the median of lymph nodes removed in contralateral lymphadenectomy [4.5 (IQR 3–7)] [9].

### 3.4. Perioperative Outcomes

Mean operative time ranged from 108 to 400 mins, while EBL ranged from 30 to 630 mL. Overall, 70 complications were described. Eight complications were classified as ≥grade III according to the Clavien–Dindo classification [9,28]: acute kidney failure (one patient), postoperative hemorrhage (one patient), hematoma at the space of Retzius (one patient), acute pulmonary edema (one patient), lymphocele (four patients). Additionally, length of hospital stay (LOS) ranged from 3 to 6 days, and catheterization time ranged from 5 to 18 days. In only one obese patient (BMI: 32 kg/m^2^), conversion to open surgery was needed [18]; thus, the surgical procedure was not completed and the patient was referred to external beam radiotherapy.

### 3.5. Oncologic Outcomes

Interestingly, a correct stratification of pT2 tumors was not always reported. One study including 16 patients reported only the percentage of each final pathological stage [13]. For this reason, an overall rate of each pathological T category was not reported. The majority of the studies included in this review did not report data on the number of lymph nodes (LN) removed in lymphadenectomy. One study reported a mean of 4.5 (IQR 3–7) LNs removed after contralateral lymphadenectomy; no LNs were found positive for PCa at the final pathological analysis [9]. Another study reported the number of LNs removed in a single bilateral lymphadenectomy (19), 3 of which were positive for PCa. Overall, 45/186 (24.19%) patients had positive surgical margins at the final pathological analysis. In total, 19/186 (10.21%) patients experienced biochemical recurrence of PCa. Last but not least, the follow-up time was highly variable among the reported studies (1–120 months).

## 4. Discussion

All the robotic procedures reported in this study were performed in patients who had kidney transplantation performed with the standard open fashion (extraperitoneal) approach. In cases of a previous robot-assisted kidney transplantation [29] or autotransplantation [30], different modifications due to the presence of intraperitoneal adherences or the eventual presence of the graft in the intraperitoneal cavity might be needed. A lack of data regarding a possible different operative time, rate of complications and PSM in this specific setting was unearthed in our review. Robotic perineal access might be theoretically a valid alternative to the standard transperitoneal approach, especially in patients who are not candidates for pelvic lymphadenectomy. In fact, abdominal trocar placement would be avoided, thus decreasing the risk of possible graft damage.

Regarding the conventional surgical steps of radical prostatectomy, all the different approaches described in the literature may theoretically be performed [31,32,33,34]. However, considering the immunosuppressive treatment and the comorbidity of these patients with a consequent high risk of postoperative erectile dysfunction due to previous kidney transplantation, a nerve-sparing approach should be carefully evaluated before submitting patients to RARP, balancing the risk of additional perioperative complications and oncological care with the presumed andrological benefit.

### 4.1. Port Placement

Kidney transplantation provides anatomical pelvic modifications due to the presence of the graft in the iliac fossa. Thus, port placement may need to be modified with the aim of preventing accidental injury to the graft and optimizing the vision of the surgical field. A standard port placement was performed successfully in 53 procedures [9,18]. In almost all of the cases, the surgical procedure was performed successfully both in the case of standard trocar dispositions or in the case of modification. Le Clerc et al. [18] reported one case of aborted prostatectomy where robotic trocars were placed in a standard fashion. In this case, an open conversion was needed due to severe obesity causing inadequate surgical field exposure. An extraperitoneal approach for RARP may be considered after a more distal placement of the ports; however, it is unusual for the majority of surgeons. In the other series, modifications were provided to ensure adequate surgical field vision and to avoid damage to the graft. The first and more intuitive modification is a more cranial placement of all the ports to increase the distance from the graft [12,21]. Alternatively, robotic ports may be shifted away from the graft [16,22] or the assistant may be placed in the opposite side [16]. Finally, the number of trocar arms may be decreased [15,19].

### 4.2. Development of the Space of Retzius

Almost all of the surgical procedures were performed using a transperitoneal approach, including the most updated and large series from Marra et al. [9]. A modified Retzius-sparing technique was used in one study aiming to avoid accidental damage to the graft [20]. In one case, an extraperitoneal approach was used with a more medial port placement [15].

### 4.3. Lymphadenectomy

Lymphadenectomy might be the most dangerous step of RARP in RTR due to the potential high risk of renal hilar damage ipsilaterally to the graft. Consequently, the use of dedicated nomograms [35,36] should be mandatory in order to make a strict selection of patients with a high risk of LN involvement. Bilateral lymphadenectomy was performed in only four patients. As reported, a limited ipsilateral lymphadenectomy was generally performed to reduce the risk of graft damage [9]. With this, no lymphadenectomy-related complications were reported. If a lymphadenectomy was indicated, it was mostly performed contralaterally. Unfortunately, the majority of the studies included in this review did not report the number of lymph nodes removed. An important point that is not assessable in our study is related to the differences in the number of LNs removed when a lymphadenectomy is performed in an RTR.

### 4.4. Perioperative Outcomes

A previous systematic review performed on the same topic (RARP in RTR) reported a 17.1% overall complication rate [8]. Recent data report a slightly lower incidence of complications in the general population (12.3%) when the procedure is carried out by an expert surgeon [37]. In our study, complication rates were highly variable among the different studies included, especially considering case reports. Only three studies reported data from >20 patients [6,10,24], showing an overall complication rate ranging from 7.4% [24] to 41% [10]. Considering the overall complication rate of RARP in this specific population, it should be noted that, due to the anatomical features of these patients, a lower rate of bilateral lymphadenectomy was performed when compared to the general population. Moreover, the results of this study indicate an operative time ranging from 108 to 400 min vs. 199.88 min, an estimated blood loss ranging from 30 to 630 mL vs. 228.2 mL, LOS ranging from 3 to 6 days vs. 3 days and catheterization time ranging from 5 to 18 days vs. 7.8 days in comparison to non-RTRs [37].

### 4.5. Oncologic Outcomes

The majority of the patients in the current study were diagnosed with low–intermediate risk PCa, probably due to the strict post-kidney transplantation follow-up protocols and genitourinary tumor screening. This point needs to be underlined when comparing oncological results to the data available for the general population. Overall, worse oncological outcomes were observed in this review, with a PSM rate of 24.19% instead of 15.7% reported by Patel et al. in a multi-institutional study on 8095 patients [38]. A biochemical recurrence rate of 10.21% was observed in our study instead of the 2.45% rate reported by Coughlin et al. in a 24-month post-RARP outcome study presented from a randomized controlled trial [39]. A possible explanation might be the more aggressive behavior of the tumor in the immunosuppressed patients rather than only the technical challenge or the scarcity of patients included in the studies.

### 4.6. Critical Reflections

A significant difference in PSA preoperative levels among the included studies is raised in this review. Sirisopana et al. reported the highest PSA preoperative levels, ranging from 10.84 ng/mL to 130 ng/mL [25]. Differently, in the other studies, the preoperative PSA level never exceeded 12 ng/mL, with the only exception of one patient with 17 ng/mL [22]. Unfortunately, a description of prostate cancer screening protocols is rarely provided in the included studies.

Interestingly, about 40% of patients were affected by ISUP 1 PCa. Probably, active surveillance (AS) was mostly avoided in RTRs due to the generally worse oncologic outcomes reported in the literature. However, a recent international multicentric analysis on 628 patients under AS for PCa observed no significant differences in tumor progression-free survival between non-RTR and RTR patients (*p* = 0.07) [40].

Further studies are needed to address several open questions. Firstly, there is no agreement on what the most appropriate lymphadenectomy template should be. Specifically, we do not know if a significant difference in the number of LNs yielded in monolateral vs. bilateral lymphadenectomy was observed and what the BCR and complication rate after each of the abovementioned procedures were. Moreover, considering the immunosuppressive treatment in RTRs and the comorbidities of patients affected by chronic renal failure, the role of NS dissection should be investigated on the basis of the potential high risk for postoperative erectile dysfunction. Finally, the potential triggers for considering an open approach instead of a robotic-assisted technique should be defined.

### 4.7. Alternative Treatment Strategies

Despite the potential difficulties due to post-transplant anatomical changes in the pelvis, radical prostatectomy is still the preferred treatment for PCa in RTR [41]. However, according to a previous systematic review of 41 studies, up to 12% of patients were treated with external beam radiotherapy (EBR), observing cancer-specific survival rates at 1, 3 and 5 years of 98.4%, 91.1% and 87.5%, respectively. A 5.4% late complication rate was recorded after EBR. The most relevant were two cases of ureteral obstruction, one case of deep venous thrombosis (DVT) involving the graft and two ureteral strictures after surgery [41].

The overall survival of patients treated with EBR did not appear to be significantly different from those submitted to radical prostatectomy (RP) in a retrospective study from a kidney transplant referral center [42]. Thus, according to the current evidence, the choice of surgical treatment instead of EBR should mostly rely on surgeons’ experience and patient counseling.

## 5. Conclusions

RARP in RTRs can be considered relatively safe and feasible. Oncological results yielded significantly worse outcomes in terms of PSM and BCR rate compared to the data available in the published studies, with the overall complication rate highly variable among the studies included. Thus, careful patient selection and counseling should be performed before considering RARP as the treatment option in RTRs. On the other hand, low graft damage during the procedure was observed in this review. Main criticisms came from different tumor screening protocols and scarce information about lymphadenectomy techniques and outcomes among the included studies. Well-designed future studies with an increased number of patients might help us better evaluate the consequences of RARP in this specific patient group.

## Figures and Tables

**Table 1:** Characteristics of studies included in the systematic review.

Study	Number of Patients	Age (Years)	OR Time (Console Time, mins)	Blood Loss (mL)	LOS (Days)	Complications (Clavien Classification)	Grade Group	Preoperative PSA	Pathologic Staging	PSM	BCR	F/U
[12] **Jhaveri** **2008**	1	54	200	400	2	0/1	2	8.5	pT2c	0/1	0/1	6 weeks
[13] **Hoda** **2010**	16	61.8 ± 8 (51–66)	108.3 ± 3.9 (88–188)	211.1 ± 87.1 (128–498)	10.1 ± 3.4 (7–18)	1/16 Prolonged hematuria	1 in 87% 2 in 13% 4 in 0%	4.7 ± 1.4	pT2a 14% pT2b 24% pT2c 62% pT3a/b 0%	1/16	0/16	28 months
[14] **Smith** **2011**	3	48 54 61	244 400 322	50 100 75	2 2 3	0/3	1	N/A	pT2c	1/3	0/3	13 months
[15] **Ghazi** **2012**	1	68	130	125	1	0/1	2	6.93	pT2b	0/1	0/1	N/A
[16] **Polcari** **2012**	7	63.3	186	N/A	1.8	3/7 Hematuria(II) Atrial fibrillation(II) Urosepsis(II)	1 in 2 2 in 4 5 in 1	6.17	pT2c in 4 pT3a in 3	2/7	1/7	16 months
[17] **Wagener** **2012**	1	71	220	300	N/A	0/1	2	12.4	pT2	0/1	0/1	9 months
[18] **Le Clerc** **2015**	12	61.92 ± 2.98	241.3 ± 35.6	587.9 ± 261.3	N/A	1/12, acute renal failure (III)	1 in 8 3 in 4	7.34	pT2b in 1 pT2c in 8 pT3a in 2	4/12	2/12	31.2 months
[19] **Moreno** **2016**	4	61.25 ± 7.76	196 ± 20.8	N/A	3.2 ± 0.9	0/4	1 in 2 2 in 1 5 in 1	7.1 ±2.8	N/A	2/4	1/4	33 ± 6.7 months
[20] **Jenjitranant** **2016**	1	73	210	250	6	0/1	3	11.53	pT2c	1/1	N/A	1 month
[21] **Plagakis** **2016**	1	60	139	190	2	0/1	2	13	pT2c	0/1	0/1	10 years
[22] **Iizuka** **2016**	3	59 60 67	163(109) 195(153) 127(80)	75 30 50	8 9 7	1/3, urinary retention (II)	3 2 2	10.6 17 8.58	pT2 pT2 pT2	0/3	1/3	24 months 23 months 8 months
[8] **Zeng** **2018**	1	65	207	500	3	1/1, superfi- cial surgical site infection (II)	5	6.65	pT3b	1/1	1/1	3 months
[23] **Mistretta** **2019**	9	60 (56–63)	160 (145–183)	100 (100–200)	4 (3–6)	1/9 (Urosepsis)	1 in 4 2 in 3 3 in 2	5.6 (5–15)	pT2a 1/9 pT2c 6/9 pT3a 1/9 pT3b 1/9	2/9	2/9	12 months
[10] **Felber** **2020**	39	62 (58–67)	180 (125−227)	150 (150−400)	4 (3;5)	16/39 Pyelonephritis, hematoma and anaemia (I-II) 4/39 Lymphoceles (IVa)	1 in 14 2 in 18 3 in 4 4 in 0 5 in 3	6.8	pT2a 5/39 pT2b 2/39 pT2c 21/39 pT3a 9/39 pT3b 2/39	5/39	3/39	47.9 months (42.3–52.5)
[24] **Léonard** **2020**	27	63.3 (43–73)	244 (120–480)	571.3 (100–1500)	5.7 (3–16)	2/27 hematoma of the Retzius space (IIIb) acute pulmonary edema (IVa)	1 in 12 2 in 8 3 in 5 4 in 0 5 in 2	8.9 (4.4–19)	pT2a 15/27 pT2b 9/27 pT2c 2/27 pT3a 1/27 pT3b 0/27	12/27	2/27	34.9 months (0.5–85.5)
[25] **Sirisopana** **2021**	5	67 64 74 66 79	365 210 210 190 210	630 300 250 150 100	13 6 8 5 7	5/5 Blood transfusion and postoperative fever	4 1 3 4 2	25.66 10.84 11.53 130 9.63	pT3b pT2a pT2c pT3b pT2a	3/5	1/5	129 months 47 months 63 months 31 months 6 months
[26] **Shahait** **2021**	14	60.2	129.7 ± 26.3	110 ± 44.6	1	0/14	1 in 4 2–3 in 9 4–5 in 1	6.9 (4–8.6)	6/14 (pT3a pT3b)	4/14	3/14	12 months
[9] **Marra** **2022**	41	60 (57–64)	210 (170– 250)	300 (200–400)	4 (2–6)	4/41 postoperative hemorrhage(IIIa) pyelonephritis(II) urinary tract infection(II) renal insufficiency(II)	1 in 11 2 in 20 3 in 4 4 in 3 5 in 1	6.5 (5.2–10.2)	pT2 29/41 pT3 11/41	7/41	2/41	42 months

OR: operating room; LOS: length of stay; N/A: not applicable; PSA: prostate-specific antigen; PSM: positive surgical margin; BCR: biochemical recurrence; F/U: follow-up.

**Table 2:** Modifications to the standard surgical technique.

Study	Number of Patients	Port Placement			Lymphadenectomy				Development of the Space of Retzius				
		Changed	No Change	Not Described	Bilateral	Contralateral	Not Performed	Not Described	No Change	Extraperitoneal	Retzius Sparing	Contralateral	Not Described
[12] **Jhaveri** **2008**	1	1	0		0	0	1		0	0	0	1	
[13] **Hoda** **2010**	16			16				16					16
[14] **Smith** **2011**	3	2	1		0	0	3		0	0	0	3	
[15] **Ghazi** **2012**	1	1	0					1	0	1	0	0	
[16] **Polcari** **2012**	7	7	0		0	4	3		0	0	0	7	
[17] **Wagener** **2012**	1	0	1		0	1	0		0	0	0	1	
[18] **Le Clerc** **2015**	12	0	12		0	12	0						12
[19] **Moreno** **2016**	4	4	0		0	0	4		0	0	0	1	3
[20] **Jenjitranant** **2016**	1	0	1		0	0	1		0	0	1	0	
[21] **Plagakis** **2016**	1	0	1		0	0	1		1	0	0	0	
[22] **Iizuka** **2016**	3	3	0		0	0	3		0	0	0	3	
[8] **Zeng** **2018**	1	1	0		0	1	0		0	0	0	1	
[23] **Mistretta** **2019**	9	9	0		1	1	0	7	0	0	9	0	
[10] **Felber** **2020**	39	0	39		1	12	26						39
[24] **Léonard** **2020**	27			27	0	12	13	2					27
[25] **Sirisopana** **2021**	5	5	0					5	5	0	0	0	
[26] **Shahait** **2021**	14	0	14					14	14	0	0	0	
[9] **Marra** **2022**	41	0	41		2	10	0	29	41	0	0	0	

**N/R**: not reported.

## Supplementary Materials

The following supporting information can be downloaded at: https://www.mdpi.com/article/10.3390/jcm12216754/s1.
