# Supplementary material for: Robot-Assisted Radical Prostatectomy in Renal Transplant Recipients: A Systematic Review
Source: J Clin Med. 2023 Oct 25;12(21):6754. doi: 10.3390/jcm12216754 (PMC10649554; doi:10.3390/jcm12216754)
Supplement: Supplementary file 1 [file jcm-12-06754-s001.zip › jcm-2611075-supplementary.pdf]

Search strings:

- **PubMed**

("Prostatic Neoplasms/surgery"[Mesh] OR "Prostatectomy"[Mesh] OR prostatect*[tiab] OR (resection[tiab] OR removal[tiab] AND prostat*[tiab])) AND ("Robotics"[Mesh] OR "Robotic Surgical Procedures"[Mesh] OR robot*[tiab] OR Davinci*[tiab] OR "Da-Vinci*"[tiab] OR Senhance*[tiab] OR "Revo-I*"[tiab] OR Versius*[tiab] OR Avatera*[tiab] OR Hinotori*[tiab]) AND ("Kidney Transplantation"[Mesh] OR (("Kidney"[Mesh] OR kidney*[tiab] OR renal[tiab] OR nephro*[tiab]) AND (allorecipient*[tiab] OR autorecipient*[tiab] OR "transplantation"[Subheading] OR "Transplantation"[Mesh] OR "Transplants"[Mesh] OR transplant*[tiab] OR allotransplant*[tiab] OR autotransplant*[tiab] OR allograft*[tiab] OR autograft*[tiab] OR graft*[tiab] OR recipient*[tiab])))

- **Embase**

('robot-assisted prostatectomy'/exp OR (('prostate tumor'/exp/dm_su OR 'prostatectomy'/exp OR prostatect*:ti,ab,kw OR (resection:ti,ab,kw OR removal:ti,ab,kw AND prostat*:ti,ab,kw)) AND ('robotics'/exp OR 'robot assisted surgery'/exp OR 'robotic surgical procedure'/exp OR 'robotic surgical device'/exp OR robot*:ti,ab,kw OR Davinci*:ti,ab,kw OR 'Da-Vinci*':ti,ab,kw OR Senhance*:ti,ab,kw OR 'Revo-I*':ti,ab,kw OR Versius*:ti,ab,kw OR Avatera*:ti,ab,kw OR Hinotori*:ti,ab,kw))) AND ('kidney transplant recipient'/exp OR 'kidney transplantation'/exp OR (('kidney'/exp OR kidney*:ti,ab,kw OR renal:ti,ab,kw OR nephro*:ti,ab,kw) AND (allorecipient*:ti,ab,kw OR autorecipient*:ti,ab,kw OR 'transplantation'/exp OR transplant*:ti,ab,kw OR allotransplant*:ti,ab,kw OR autotransplant*:ti,ab,kw OR allograft*:ti,ab,kw OR autograft*:ti,ab,kw OR graft*:ti,ab,kw OR recipient*:ti,ab,kw)))

- **Scopus**

(((TITLE-ABS-KEY (prostatect*)) OR (TITLE-ABS-KEY (resection OR removal AND prostat*))) AND (TITLE-ABS-KEY (robot* OR davinci* OR "Da-Vinci*" OR senhance* OR "Revo-I*" OR versius* OR avatera* OR hinotori*))) AND ((TITLE-ABS-KEY (kidney* OR renal OR nephro*)) AND (TITLE-ABS-KEY (allorecipient* OR autorecipient* OR transplant* OR allotransplant* OR autotransplant* OR allograft* OR recipient*)))
